# Effect of Saturated Stearic Acid on MAP Kinase and ER Stress Signaling Pathways during Apoptosis Induction in Human Pancreatic β-Cells Is Inhibited by Unsaturated Oleic Acid

**DOI:** 10.3390/ijms18112313

**Published:** 2017-11-02

**Authors:** Jan Šrámek, Vlasta Němcová-Fürstová, Nela Pavlíková, Jan Kovář

**Affiliations:** Division of Cell and Molecular Biology & Center for Research of Diabetes, Metabolism and Nutrition, Third Faculty of Medicine, Charles University, Ruská 87, 100 00 Prague, Czech Republic; jan.sramek@lf3.cuni.cz (J.Š.); vlasta.furstova@centrum.cz (V.N.-F.); nela.pavlikova@lf3.cuni.cz (N.P.)

**Keywords:** fatty acids, pancreatic β-cells, apoptosis, p38 mitogen-activated protein kinase (MAPK), extracellular signal-regulated kinase (ERK), endoplasmic reticulum (ER) stress, NES2Y

## Abstract

It has been shown that saturated fatty acids (FAs) have a detrimental effect on pancreatic β-cells function and survival, leading to apoptosis, whereas unsaturated FAs are well tolerated and are even capable of inhibiting the pro-apoptotic effect of saturated FAs. Molecular mechanisms of apoptosis induction and regulation by FAs in β-cells remain unclear; however, mitogen-activated protein (MAP) kinase and endoplasmic reticulum (ER) stress signaling pathways may be involved. In this study, we tested how unsaturated oleic acid (OA) affects the effect of saturated stearic acid (SA) on the p38 mitogen-activated protein kinase (MAPK) and extracellular signal-regulated kinase (ERK) pathways as well as the ER stress signaling pathways during apoptosis induction in the human pancreatic β-cells NES2Y. We demonstrated that OA is able to inhibit all effects of SA. OA alone has only minimal or no effects on tested signaling in NES2Y cells. The point of OA inhibitory intervention in SA-induced apoptotic signaling thus seems to be located upstream of the discussed signaling pathways.

## 1. Introduction

Increased levels of fatty acids (FAs) in blood are considered to be one of the main factors responsible for pancreatic β-cell death in type 2 diabetes [1,2,3,4,5]. Our previous studies as well as other studies have shown that this effect of FAs is related to the saturation of particular FAs. Saturated FAs (palmitic or stearic acid) have detrimental effects on pancreatic β-cells survival, leading to apoptosis, whereas unsaturated FAs (oleic or palmitoleic acid) are well tolerated and even capable of inhibiting the pro-apoptotic effect of saturated FAs [2,4,5,6,7,8]. Molecular mechanisms of apoptosis induction and its regulation by FAs in β-cells remain unclear [9]. However, it was shown that apoptosis induced by saturated FAs can be mediated by p38 MAPK (mitogen-activated protein kinase) and ERK (extracellular signal-regulated kinases) MAPK signaling pathways [10,11,12,13,14] as well as by endoplasmic reticulum (ER) stress signaling [15,16].

The p38 MAPK pathway becomes mainly activated by the dual-specific MKK3/6 (mitogen-activated protein kinase kinase 3 and 6) kinase due to various extracellular stimuli such as chemical stresses (reviewed in [17]). p38 MAPK regulates the activity of MAPKAPK-2 (MAPK-activated protein kinase 2) which is involved in nuclear export of activated p38 MAPK [18]. It may also affect the activation of some proteins such as NF-κB (nuclear factor kappa B) [19] or caspase-3 [20]. These proteins are involved in the regulation of apoptosis induction.

The ERK pathway is mostly activated by growth factors. Growth factors, acting through receptor kinases and adaptor protein son of sevenless (SOS), activate Ras GTPase, which is responsible for c-Raf phosphorylation [21]. This kinase consequently phosphorylates MEK1/2 (mitogen-activated protein kinase/ERK kinase), which leads to the phosphorylation and thus activation of ERK1/2 kinase. Like p38 MAPK, ERK1/2 can affect the activation of various proteins that also regulate apoptosis, such as Fox03a (forkhead box O3a) or several proteins of the Bcl-2 (B-cell lymphoma) family [22]. 

ER stress signaling is represented by three known pathways. Their activation seems to begin on the ER membrane by the activation of three proteins: (1) IRE1α (inositol-requiring protein 1α); (2) PERK (protein kinase RNA (PKR)-like ER kinase); and (3) ATF6 (activating transcription factor 6). Activated IRE1α causes unconventional splicing of mRNA for XBP1 (X-box binding protein 1), which leads to the translation of active transcription factor (XBP1s). It also leads to JNK (c-Jun N-terminal kinase) activation by phosphorylation, which further phosphorylates c-Jun. Activated PERK results in the inhibition of protein translation via eIF2α (eukaryotic initiation factor 2α) phosphorylation. ATF6 translocation to the nucleus, where ATF6 functions as a transcription factor, represents the activation of the ATF6 pathway. The main purpose of these signaling patterns is to restore ER homeostasis, e.g., by decreasing protein translation and increasing the expression of chaperones, such as the ER chaperone BiP (immunoglobulin heavy chain-binding protein) [23]. However, failure of these responses leads to apoptosis induction by not fully clear mechanisms. The proposed mediator is transcription factor CHOP (CCAAT-enhancer-binding protein (C/EBP) homologous protein) [24].

In the present study, we tested how unsaturated OA affects the effect of saturated SA on the p38 MAPK and ERK pathways as well as the ER stress signaling pathways during apoptosis induction in the human pancreatic β-cells NES2Y. We demonstrate that OA is able to inhibit all effects of SA. OA alone had minimal or no effects on tested signaling in NES2Y cells. The point of OA inhibitory intervention in SA-induced apoptotic signaling thus seems to be located upstream of the discussed signaling pathways.

## 2. Results

### 2.1. Effect of Oleic Acid on Stearic Acid-Induced Apoptosis

**Figure 1:**
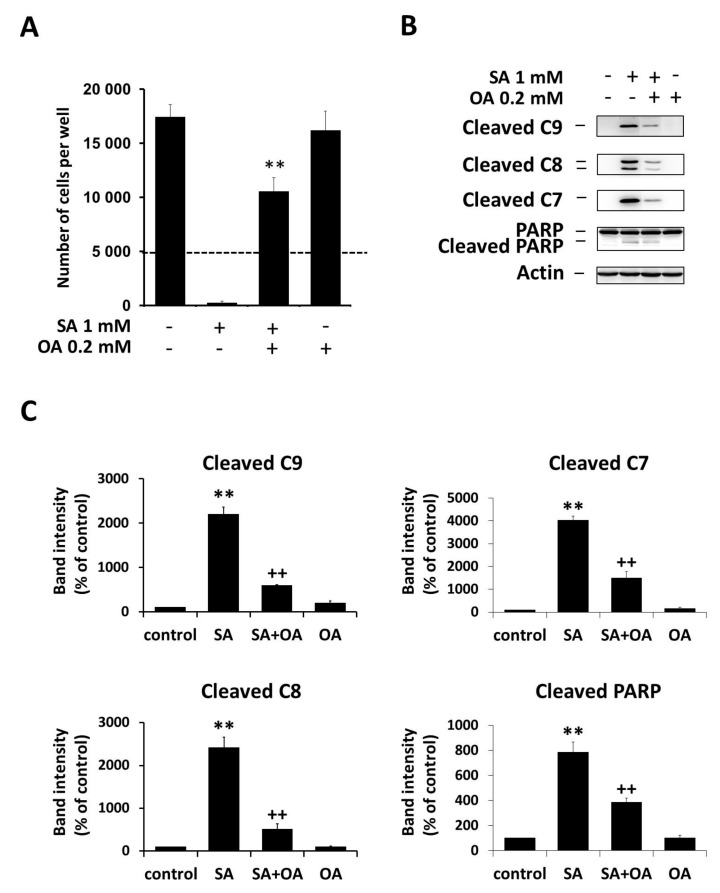
Effect of 1 mM stearic acid (SA), 1 mM stearic acid applied simultaneously with 0.2 mM oleic acid (OA), and 0.2 mM oleic acid (see Section 4) on (**A**) cell growth and viability, (**B**) the level of cleaved caspase-9 (C9), caspase-8 (C8), caspase-7 (C7), and PARP in NES2Y cells. Cells incubated without fatty acids represented control cells. (**A**) Cells were seeded at a concentration of 5 × 10^3^ cells/100 µL of culture medium per well of 96-well plate (see Section 4). The number of living cells was determined after 96 h of incubation. The number of cells of the inoculum is shown as a dashed line. Each column represents the mean of four separate cultures ± standard error of the mean (SEM). ** *p* < 0.01 when comparing the effect of 1 mM stearic acid applied together with 0.2 mM oleic acid and the effect of 1 mM stearic acid alone. (**B**) After 18 h of incubation (see Section 4), the levels of individual proteins were assessed using Western blot analysis and relevant antibodies (see Section 4). Actin was included to confirm equal protein loading. The data presented were obtained in one representative experiment from at least three independent experiments. (**C**) Densitometric analysis of data from Western blotting are also shown. Each column represents the mean of three experimental values ± SEM. ** *p* < 0.01 when comparing the effect of SA with control cells, ^++^ *p* < 0.05 when comparing the effect of SA plus OA with the effect of SA alone.

We showed that OA (0.2 mM) inhibited SA-induced (1 mM) cell death of NES2Y cells. OA applied alone has no or only minimal effects on NES2Y cells (Figure 1A). Cell death induced by SA is associated with the activation of initiator caspase-9 and -8 as well as executioner caspase-7 and poly ADP-ribose polymerase (PARP) (substrate of caspase-7) cleavage. Thus, it can be characterized as apoptosis. The activation of caspases as well as PARP cleavage is inhibited by the co-incubation of OA (Figure 1B,C). Cleavage of caspase-2 and -3 was not tested, since we found previously that there is nearly no activation of caspase-3 in NES2Y cells after SA exposure, and that caspase-2 does not play a key role in SA-induced apoptosis [8,25].

### 2.2. Effect of Oleic Acid on the Activation of the p38 MAPK and the Inhibition of the ERK Signaling Pathways by Stearic Acids

We previously showed the activation of the p38 MAPK signaling pathway and the inhibition of the ERK signaling pathway within 24 h of the application of apoptosis-inducing SA (1 mM) in NES2Y β-cells [14]. In order to test whether OA (0.2 mM) could interfere with the effect of SA, we assessed the activation of the p38 MAPK signaling pathway and inhibition of the ERK signaling pathway within 24 h after OA co-incubation with SA in NES2Y cells.

**Figure 2:**
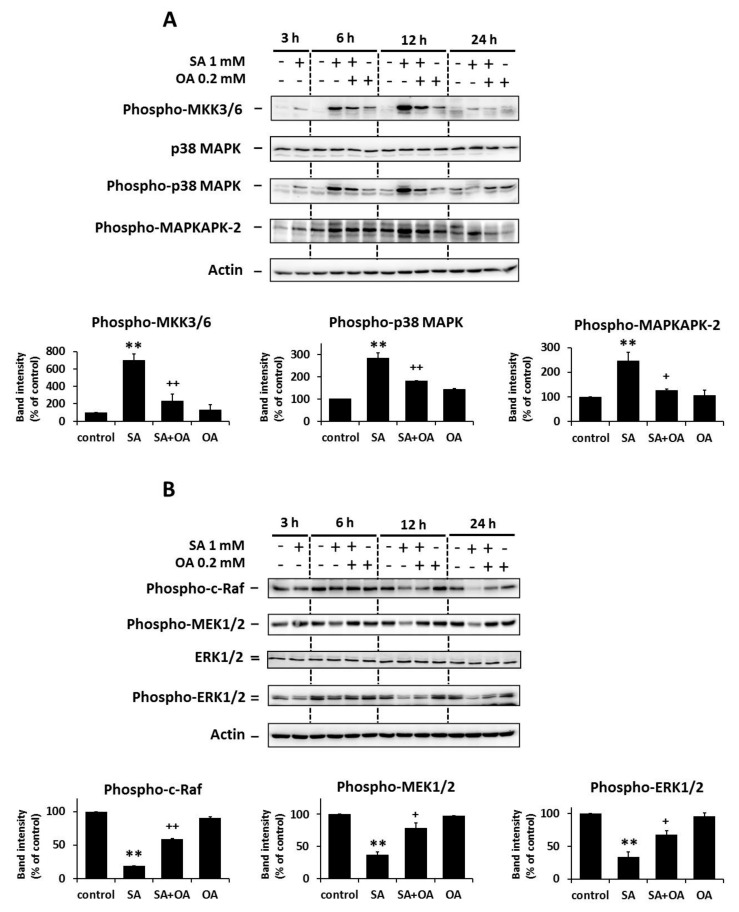
Effect of 1 mM stearic acid (SA), 1 mM stearic acid applied simultaneously with 0.2 mM oleic acid (OA), and 0.2 mM oleic acid (see Section 4) on (**A**) the levels of phospho-MKK3/6, p38 MAPK, phospho-p38 MAPK, and phospho-MAPKAPK-2 (the p38 MAPK signaling pathway); and (**B**) the levels of phospho-c-Raf, phospho-MEK1/2, ERK1/2, and phospho-ERK1/2 (the ERK signaling pathway) in NES2Y cells. Cells incubated without fatty acids represented control cells. After 3, 6, 12, and 24 h of incubation (see Section 4), the levels of individual proteins were assessed using Western blot analysis and relevant antibodies (see Section 4). Actin was included to confirm equal protein loading. The data presented were obtained in one representative experiment from at least three independent experiments. Densitometric analysis of data from Western blotting is also shown. The analysis was carried out for 12 h after fatty acids application in the case of the p38 MAPK pathway and for 24 h in the case of the ERK pathway. Each column represents the mean of three experimental values ± SEM. ** *p* < 0.01 when comparing the effect of SA with control cells, ^+^ *p* < 0.05, ^++^ *p* < 0.01 when comparing the effect of SA plus OA with the effect of SA alone.

The effect of SA on the activation of the p38 pathway members, i.e., an increase in the level of phosphorylated MKK3/6, p38 MAPK, and MAPKAPK-2, was inhibited by co-incubation with OA as early as 12 h after the treatment in all of the tested proteins. OA applied alone resulted in low or nearly no changes in the level of phosphorylated p38 MAPK pathway members. No change was detected in the level of total p38 MAPK within 24 h of FA treatment (Figure 2A).

The effect of SA on the inhibition of the ERK pathway members, i.e., a decrease in the level of phosphorylated c-Raf, MEK1/2, and ERK1/2, was also inhibited by OA co-incubation within 24 h of the treatment. Separate application of OA resulted in nearly no effect on the level of phosphorylated ERK pathway members. We did not detect any change in the level of total ERK1/2 within 24 h of FA treatment (Figure 2B).

### 2.3. Effect of Oleic Acid on the Activation of the ER Stress Signaling Pathways by Stearic Acid

We previously documented the activation of ER stress signaling pathways (IRE1α and PERK pathways) within 24 h of apoptosis-inducing SA (1 mM) treatment in NES2Y β-cells [25]. In order to test whether OA (0.2 mM) could affect the SA-induced activation of ER stress signaling pathways, we assessed the activation of members of the ER stress IRE1α pathway (the levels of phospho-IRE1α, phospho-JNK, phospho-c-Jun) and the PERK pathway (the level of phospho-eIF2α) within 24 h after OA co-incubation with SA in NES2Y cells. The level of XBP1 splicing as well as the level of two downstream effector molecules, CHOP and BiP (known as ER stress markers), were also tested.

**Figure 3:**
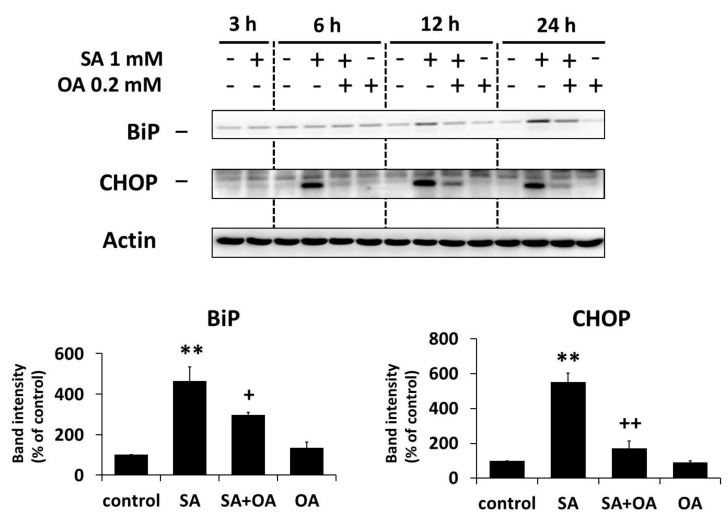
Effect of 1 mM stearic acid (SA), 1 mM stearic acid applied simultaneously with 0.2 mM oleic acid (OA), and 0.2 mM oleic acid (see Section 4) on the level of ER stress markers BiP and CHOP in NES2Y cells. Cells incubated without fatty acids represented control cells. After 3, 6, 12, and 24 h of incubation (see Section 4), the levels of individual proteins were assessed using Western blot analysis and relevant antibodies (see Section 4). Actin was included to confirm equal protein loading. The data presented were obtained in one representative experiment from at least three independent experiments. Densitometric analysis of data from Western blotting is also shown. The analysis was carried out for 24 h after fatty acids application. Each column represents the mean of three experimental values ± SEM. ** *p* < 0.01 when comparing the effect of SA with control cells, ^+^ *p* < 0.05, ^++^ *p* < 0.01 when comparing the effect of SA plus OA with the effect of SA alone.

**Figure 4:**
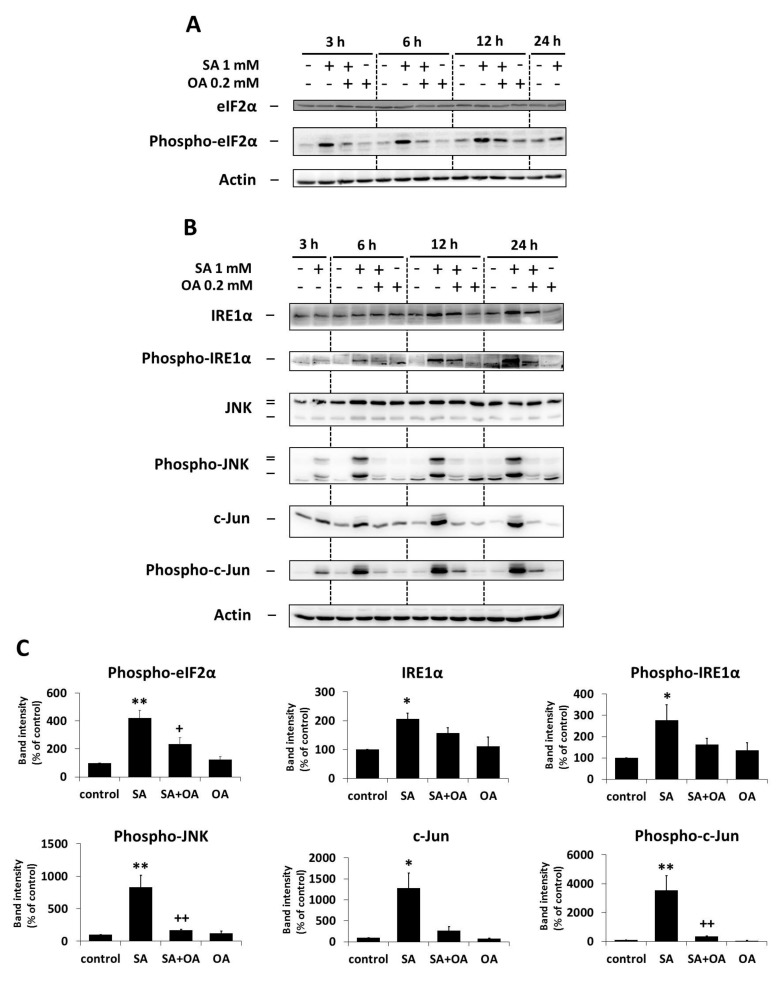
Effect of 1 mM stearic acid (SA), 1 mM stearic acid applied simultaneously with 0.2 mM oleic acid (OA), and 0.2 mM oleic acid (see Section 4) on the levels of (**A**) eIF2α and phospho-eIF2α, and (**B**) IRE1α, phospho-IRE1α, JNK, phospho-JNK, c-Jun, and phospho-c-Jun in NES2Y cells. Cells incubated without fatty acids represented control cells. After 3, 6, 12, and 24 h of incubation (see Section 4) (**A**,**B**), the levels of individual proteins were assessed using Western blot analysis and relevant antibodies (see Section 4). Actin was included to confirm equal protein loading. (**C**) Densitometric analysis of data from Western blotting is also shown. The analysis was carried out for 6 h after fatty acids application in the case of phospho-eIF2α, for 12 h in the case of phospho-JNK, c-Jun and phospho-c-Jun, and for 24 h in the case of IRE1α and phospho-IRE1α. Each column represents the mean of three experimental values ± SEM. * *p* < 0.05, ** *p* < 0.01 when comparing the effect of SA with control cells. ^+^ *p* < 0.05, ^++^ *p* < 0.01 when comparing the effect of SA plus OA with the effect of SA alone.

**Figure 5:**
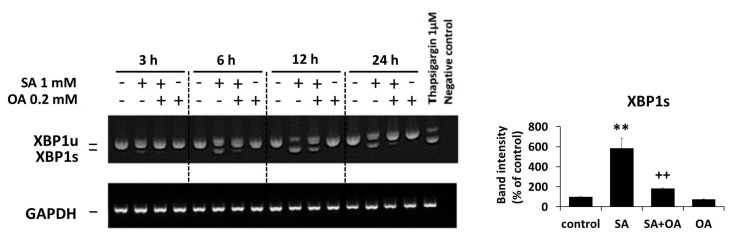
Effect of 1 mM stearic acid (SA), 1 mM stearic acid applied simultaneously with 0.2 mM oleic acid (OA), and 0.2 mM oleic acid (see Section 4) on XBP1 splicing in NES2Y cells. Cells incubated without fatty acids represented control cells. After 3, 6, 12, and 24 h of incubation (see Section 4), the XBP1 splicing was assessed by RT-PCR using relevant primers (see Section 4). NES2Y cells were treated with 1 μM thapsigargin as a positive control of XBP1 splicing. GAPDH (glyceraldehyde-3-phosphate dehydrogenase) was used as a control gene for RT-PCR. The data presented were obtained in one representative experiment from at least three independent experiments. Densitometric analysis of data from RT-PCR is also shown. The analysis was carried out for 24 h after fatty acids application. Each column represents the mean of three experimental values ± SEM. ** *p* < 0.01 when comparing the effect of SA with control cells, ^++^ *p* < 0.01 when comparing the effect of SA plus OA with the effect of SA alone.

The effect of SA on the activation of ER stress pathways, i.e., an increase in the levels of phospho-IRE1α (and IRE1α), phospho-JNK (JNK level was not changed), phospho-c-Jun (and c-Jun), phospho-eIF2α (eIF2α level was not changed), CHOP and BiP, as well as the induction of XBP1 splicing, was significantly inhibited by OA co-incubation within 24 h of the treatment. Separate application of OA resulted in nearly no changes in the level of tested proteins and XBP1 splicing (Figure 3, Figure 4 and Figure 5).

## 3. Discussion

We, as well as other authors, have shown that unsaturated FAs have nearly no detrimental effect on pancreatic β-cells, but that they are in fact capable of inhibiting the pro-apoptotic effect of saturated FAs [2,4,5,6,7,8]. The molecular mechanisms involved in the modulation of saturated FAs-induced apoptosis by unsaturated FAs in β-cells are not fully clear. However, it seems that the effect of unsaturated FAs is mediated by the regulation of some signaling pathways rather than by their direct interference with the metabolism of saturated FAs [6,26,27]. Therefore, in this paper we tested the modulation of the effects of apoptosis-inducing saturated SA on the p38 MAPK pathway, the ERK pathway, and also on ER stress signaling pathways IRE1α and PERK by unsaturated OA.

Our data showed that the activation of the p38 MAPK pathway, inhibition of the ERK pathway, and activation of the IRE1α and PERK ER stress pathways by SA were inhibited by OA (see Figure 2, Figure 3, Figure 4 and Figure 5). The inhibitory effect of OA on the effect of saturated FAs on the p38 MAPK and ERK pathways has not been demonstrated, to our knowledge, until now. On the other hand, the inhibitory effect of OA on palmitate-induced ER stress was already documented in BRIN-BD11 and INS-1E rat pancreatic β-cells [7,28]. However, our study concerning the effect of SA presented more data. 

We also provided a pilot experiment (using confocal microscopy), in which we tested the effect of OA on the SA-induced activation of the ATF6 pathway of ER stress signaling. We found (data not shown) that SA-induced ATF6 activation and translocation into the nucleus did not seem to be inhibited by OA co-incubation. Separate administration of OA had no effect on ATF6 translocation. However, this finding was not confirmed by an independent method.

Our findings concerning both MAP kinase signaling pathways as well as ER stress signaling pathways support the fact that the point of OA inhibitory intervention in SA-induced apoptotic signaling is located upstream of this signaling. Our preliminary data, as well as some previous studies [29,30], indicate that the point of intervention may be located on the plasma membrane where FAs affect membrane fluidity [31]. We hypothesize that saturated FAs with rigid and straight acyl chain conformation reduces membrane fluidity after incorporation into the lipid bilayer. This could alter the capability of membrane receptor(s) (receptor tyrosine kinases) to dimerize and thus transfer signals. Signals in the case of receptor tyrosine kinases can be signals for the stimulation of proliferation and/or pro-survival signals. Concerning a possible mechanism of OA inhibitory intervention in SA signaling leading to apoptosis induction, we speculate that the inhibitory effect of OA can be generated simply by increasing membrane fluidity, which compensate for the effect of SA. 

In conclusion, we have shown, employing human pancreatic β-cells NES2Y, that unsaturated OA is able to inhibit the effects of saturated SA on the activation of the p38 MAPK signaling pathway and inhibition of the ERK signaling pathway, as well as activation of the IRE1α and PERK ER stress signaling pathways. OA alone had minimal or no effects on tested signaling in NES2Y cells. The point of OA inhibitory intervention in SA-induced apoptotic signaling thus seems to be located upstream of the discussed signaling pathways.

## 4. Materials and Methods

### 4.1. Materials

Chemicals were from Sigma-Aldrich (St. Louis, MO, USA), unless otherwise stated. The following primary and secondary antibodies for Western blot analysis were used: anti-phospho-MKK3/6 (#9236), anti-p38 MAPK (#8690), anti-phospho-p38 MAPK (#4511), anti-phospho-MAPKAPK-2 (#3007), anti-phospho-c-Raf (#9427), anti-phospho-MEK1/2 (#9154), anti-ERK1/2 (#5013), anti-phospho-ERK1/2 (#4370), anti-BiP (#3177), anti-CHOP (#2895), anti-eIF2α (#9722), anti-phospho-eIF2α (#9261), anti-IRE1α (#3294), anti-SAPK/JNK (#9258), anti-phospho-SAPK/JNK (#4668), anti-c-Jun (#9165) and anti-phospho-c-Jun (#9261), anti-PARP (#9542), anti-cleaved caspase-7 (#9491), anti-cleaved caspase-8 (#9496), anti-cleaved caspase-9 (#9505) from Cell Signaling Technology (Danvers, MA, USA), anti-phospho-IRE1α (ab 48187) and anti-GAPDH (ab9485) from Abcam (Cambridge, UK), and anti-actin (clone AC-40).

### 4.2. Cells and Culture Conditions

The human pancreatic β-cell line NES2Y employed in the experiments was kindly provided by Roger F. James (Department of Infection, Immunity and Inflammation, University of Leicester) [5,32]. NES2Y cells are proliferating cells secreting insulin with a defect in glucose responsiveness. Cells were regularly maintained in an RPMI 1640-based culture medium [33]. A defined serum-free medium [34] supplemented with FAs (1 mM SA, a combination of 1 mM SA and 0.2 mM OA, or 0.2 mM OA alone) bound to a 2% FA-free bovine serum albumin (BSA) was used in experiments [5]. Stock solutions containing FAs bound to 10% BSA in a serum-free medium were made as described previously [5] and diluted to the required concentration of FA and BSA prior to experiments. Molar ratios of FA/BSA used in experiments were lower than the ratios known to exceed the binding capacity of BSA [35].

Our previous studies showed that SA, at a concentration of 1 mM, leads to the activation of the p38 MAPK and ER stress signaling pathways and to the inhibition of the ERK signaling pathway within 24 h of the treatment [8,14]. Therefore, all assessments were performed within 24 h of treatment, except for the assessment of cell growth and viability. Since the physiological concentration of SA in adult serum seems to vary between 0.110–1.170 mM [36,37], we used 1 mM concentration of SA to simulate an elevated level of SA. An increased level of circulating FAs is a common characteristic of obese individuals [38], and a connection between obesity and type 2 diabetes mellitus (in addition to insulin resistance) has been clearly presented [38,39]. Finally, 0.2 mM concentration of OA was used, since this was the lowest concentration sufficient to inhibit the detrimental effects of SA [8].

### 4.3. Assessment of the Effect of Oleic Acid on the Effects of Stearic Acid on Cell Growth and Viability

Cells were seeded into the wells of a 96-well plate at a concentration of 5 × 10^3^ cells/100 μL of culture media. After a 24-h pre-incubation period (allowing cells to attach) the culture medium was substituted with a serum-free medium containing 2% BSA with or without fatty acid(s). The control medium contained 2% BSA only. After 96 h of incubation, the number of living cells was assessed using a hemocytometer counting system, after staining with trypan blue.

### 4.4. Western Blot Analysis

Cells (approximately 1 × 10^6^ cells per sample) were seeded and, after a 24-h pre-incubation period (allowing cells to attach), the culture medium was substituted with a serum-free medium containing 2% BSA with or without FA(s) (SA, a combination of SA and OA, or OA alone) at required concentrations. The control medium contained 2% BSA only. After the required incubation period, cells were harvested and Western blot analysis was performed as described previously [8]. All primary antibodies were used in a 1:1000 dilution. The chemiluminescent signal was detected using a Carestream Gel Logic 4000 PRO Imaging System equipped with Carestream Molecular Imaging Software (Carestream Health, New Haven, CT, USA), which was used for image acquisition. Image Master™ 2D Platinum 6.0 software (GE Healthcare, Uppsala, Sweden) was used to obtain data for densitometric analyses.

### 4.5. Assessment of XBP1 mRNA Splicing 

Cells (approximately 1 × 10^6^ cells per sample) were seeded and, after 24 h pre-incubation, FAs and thapsigargin were applied as described above (see Section 4.4). After 3, 6, 12, and 24 h incubation, the cells were harvested and the splicing of XBP1 mRNA was ascertained by RT-PCR as described previously [8,40] using GAPDH as a housekeeping gene [41].

### 4.6. Statistical Analysis

The statistical significance of observed differences was determined using the Tukey test.

## Abbreviations

ATF6	Activating transcription factor 6
Bcl-2	B-cell lymphoma
BiP	Immunoglobulin heavy chain-binding protein
CHOP	CCAAT-enhancer-binding protein (C/EBP) homologous protein
eIF2α	Eukaryotic initiation factor 2α
ERK1/2	Extracellular signal-regulated kinase 1/2
ER	Endoplasmic reticulum
FA	Fatty acid
Foxo03a	Forkhead box 03a
GAPDH	Glyceraldehyde-3-phosphate dehydrogenase
IRE1α	Inositol-requiring protein 1
JNK	c-Jun N-terminal kinase
MAP	Mitogen-activated protein
MAPK	Mitogen-activated protein kinase
MAPKAPK	MAP kinase-activated protein kinase
MEK1/2	Mitogen-activated protein kinase/ERK kinase
NF-κB	Nuclear factor kappa B
PARP	Poly ADP-ribose polymerase
PERK	Protein kinase RNA (PKR)-like ER kinase
SA	Stearic acid
SEM	Standard error of the mean
SOS	Son of sevenless
TG	Thapsigargin
T2DM	Type 2 diabetes mellitus
OA	Oleic acid
XBP1	X-box binding protein 1
